# Pathology-informed Generative Adversarial Network Augmentation Improves Classification of Peripheral Nerve Sheath Tumors by Modeling Morphological Variability: A Pilot Investigation

**DOI:** 10.1007/s12105-026-01939-7

**Published:** 2026-06-26

**Authors:** Giovanna Calabrese dos Santos, Hyago Vieira Lemes Barbosa Silva, Anna Luíza Damaceno Araújo, Thaís Cerqueira Reis Nakamura, Micael Gil Scholl Santos, Anderson Faria Claret, Felipe Augusto Pereira de Figueiredo, Sebastião Silvério de Sousa-Neto, Daniela Giraldo-Roldán, Leonardo Marcello Rener de Freitas, Luiz Paulo Kowalski, Alan Roger Santos-Silva, Marcio Ajudarte Lopes, Pablo Agustin Vargas, Matheus Cardoso Moraes

**Affiliations:** 1 Institute of Science and Technology, Federal University of São Paulo (ICT- UNIFESP), São José dos Campos, São Paulo, Brazil; 2 National Institute of Telecommunications - INATEL, Santa Rita Do Sapucaí, MG Brazil; 3 Hospital Israelita Albert Einstein, Av. Professor Francisco Morato, 4293, Butantã, São Paulo, 05521-200 Brazil; 4 Departamento de Diagnóstico Oral, Faculdade de Odontologia de Piracicaba, Universidade Estadual de Campinas (FOP-UNICAMP), Piracicaba, São Paulo, Brazil; 5 Head and Neck Surgery Department, University of São Paulo Medical School, São Paulo, Brazil; 6 Department of Head and Neck Surgery and Otorhinolaryngology, A.C. Camargo Cancer Center, São Paulo, Brazil

**Keywords:** Peripheral nerve sheath tumors, Generative adversarial networks, Convolutional neural networks, Image augmentation, Perineurioma, Whole-slide images, Synthetic patch generation

## Abstract

**Background:**

Peripheral nerve sheath tumors (PNSTs) of the head and neck (H&N) show histopathological overlap. Although convolutional neural networks (CNNs) have demonstrated feasibility in soft tissue tumor classification, limited intra-class variability related to perineurioma remains a critical constraint for rare tumor subtypes.

**Methods:**

This retrospective diagnostic accuracy study with internal validation included 30 patients diagnosed with PNSTs. Whole-slide images were digitized at 20× magnification and partitioned using a strict patient-wise split. Synthetic perineurioma patches were generated using a modified Pix2Pix-based Generative Adversarial Network (GAN) incorporating a bottleneck architecture and self-attention modules. Two morphology-driven augmentation strategies were evaluated: (1) intra-phenotypic expansion by cross-patient patch pairing within the sclerosing subtype and (2) inter-phenotypic interpolation by cross-phenotype patch pairing between sclerosing and intraneural variants. EfficientNetV2-B0 pre-trained on ImageNet was trained under three configurations: original dataset only, original + Experiment A synthetic patches, and original + Experiment B synthetic patches. All performance metrics were computed exclusively on an independent yet internal test set composed of original images.

**Results:**

GAN-based augmentation improved global classification performance compared with the baseline model trained on original images only (accuracy 0.733). Intra-phenotypic expansion increased accuracy to 0.767 and achieved the highest balanced accuracy (0.750) and macro-F1 (0.740). Inter-phenotypic interpolation yielded the highest overall accuracy and competitive multiclass agreement metrics. Perineurioma recall improved from 0.34 (baseline) to 0.51 with intra-phenotypic augmentation and 0.47 with inter-phenotypic interpolation, while specificity remained ≥ 0.999 across all strategies.

**Conclusions:**

Structured, pathology-informed GAN augmentation improved CNN classification of PNSTs, particularly for the morphologically heterogeneous perineurioma class. Intra-phenotypic expansion primarily improved rare-class sensitivity, whereas inter-phenotypic interpolation improved multiclass agreement and global robustness. These findings support morphology-driven synthetic enrichment as a clinically meaningful strategy to improve AI performance in underrepresented tumor entities and potentially support diagnostic decision-making in digital pathology environments.

## Introduction

Peripheral nerve sheath tumors (PNSTs) comprises a heterogeneous group of neoplasms derived from the cellular components of the peripheral nervous system, including Schwann cells, perineurial cells, and fibroblasts [1, 2]. In the head and neck (H&N) region, neurofibroma, perineurioma, and schwannoma represent the most frequently encountered benign entities. Although these tumors are typically distinguishable by classical histopathological criteria, overlapping morphological features may occur, particularly in small biopsies, fragmented specimens, or cases with atypical stromal architecture [1, 3].

Perineurioma poses a particular diagnostic challenge. Its morphological spectrum, including sclerosing and intraneural variants, may mimic other spindle cell neoplasms or overlap with neurofibroma and schwannoma [2, 4]. In such scenarios, immunohistochemical (IHC) analysis is often required to confirm perineurial differentiation, typically using markers such as EMA and claudin-1. However, IHC increases diagnostic cost, processing time, and dependency on specialized laboratory resources, which may not be universally available [5].

Recent advances in digital pathology and artificial intelligence (AI) have enabled the development of convolutional neural networks (CNNs) for histopathological image classification [6, 7]. CNN-based approaches have demonstrated feasibility in soft tissue tumor recognition and H&N pathology applications [8–10]. In a previous investigation conducted by our group, CNN architectures, specifically ResNet50, achieved satisfactory performance in distinguishing neurofibroma and schwannoma; however, perineurioma demonstrated substantially lower sensitivity, reflecting its morphological heterogeneity and relative underrepresentation within the dataset [9]. This limitation was further observed across different convolutional neural network architectures explored in subsequent experiments by our group, including more recent and higher-capacity models. Despite improvements in overall performance, the sensitivity for perineurioma remained consistently lower compared to the other classes, even when using more advanced architectures that achieved the best performance (EfficientNetV2-B0) within our internal subsequent evaluations, reinforcing the hypothesis that the primary challenge lies not in model architecture, but in the limited representation of intra-class morphological variability.

Data scarcity remains a critical limitation in rare or morphologically heterogeneous tumors. Deep learning models require sufficient intra-class variability to learn robust and generalizable decision boundaries. When limited cases are available, particularly for underrepresented categories such as perineurioma, models may overfit to dominant stromal patterns and fail to generalize adequately across phenotypic variants [11]. In perineurioma, this limitation is further amplified by the coexistence of distinct architectural variants, such as sclerosing and intraneural forms, which may occupy different regions of the morphological spectrum and increase diagnostic overlap with other spindle cell tumors.

Traditional data augmentation techniques, including geometric transformations and color perturbations, increase sample volume but do not introduce novel morphological information. In contrast, Generative Adversarial Networks (GANs) have emerged as powerful generative models capable of synthesizing realistic histopathological images by modeling complex structural and textural patterns [12–14]. In digital pathology, GANs have been applied to tasks such as stain normalization, virtual staining, domain adaptation, and synthetic data augmentation, demonstrating potential to improve classifier performance in limited datasets [12, 15, 16].

Recent studies have shown that GAN-based synthetic augmentation can enhance histopathological classification performance when carefully integrated into the training pipeline [14, 17]. Moreover, representation space interpolation strategies allow the generation of transitional morphological representations between distinct phenotypic poles, effectively modeling continuous variability within a disease spectrum [18]. Such approaches may be particularly relevant for tumors with subtle morphological gradients, where diagnostic uncertainty frequently arises from overlapping architectural patterns. Conceptually, this strategy aligns with routine histopathological practice, in which pathologists often recognize intermediate or borderline morphological patterns rather than strictly discrete diagnostic categories.

Despite the increasing adoption of GAN-based methods in computational pathology, their application to PNSTs in the H&N region remains largely unexplored. In particular, it remains unclear whether synthetic image generation strategies that explicitly incorporate morphological relationships between histological variants can improve CNN-based classification performance in this tumor group.

Therefore, the present study investigates whether structured GAN-based synthetic augmentation can enhance CNN classification of neurofibroma, perineurioma, and schwannoma in digitized histopathological slides. Specifically, we evaluate two morphology-driven synthetic generation strategies: (1) intra-phenotypic expansion aimed at increasing variability within a single perineurioma subtype, and (2) inter-phenotypic interpolation designed to model transitional morphology between sclerosing and intraneural variants. The impact of these strategies is assessed on EfficientNetV2-B0 performance under a strictly controlled training protocol in which validation and internal testing are independently performed exclusively on original, non-synthetic images.

## Materials and Methods

### Case Selection and Slide Digitization

This retrospective diagnostic study with internal validation included 30 patients diagnosed with benign PNSTs. Cases were retrieved from the archives of the Faculdade de Odontologia de Piracicaba (FOP-UNICAMP). Diagnoses were confirmed based on established histopathological criteria and categorized as follows: neurofibroma (Class 0; *n* = 9), perineurioma (Class 1; *n* = 7), and schwannoma (Class 2; *n* = 14). All slides were digitized using the Aperio CS Digital Pathology System (Leica Biosystems, Wetzlar, Germany) at 20× magnification, corresponding to a spatial resolution of 0.47 μm/pixel. This study was performed in accordance with the Declaration of Helsinki and was approved on November 14, 2023, by the Faculdade de Odontologia de Piracicaba Ethics Committee (Registration number: 70281123.8.0000.5418), which also included Material Transfer Agreements between co-participant Institutions for sharing digital slides.

### Image Annotation

Whole-slide images (WSIs) were visually inspected and annotated to define regions of interest (ROIs) corresponding to tumor parenchyma. This step aimed to exclude regions unlikely to contribute to the diagnostic discrimination of the studied lesions, such as extensive stromal components common to multiple tumor types. Additionally, noisy areas, including folded tissue, staining artifacts, and blank regions without histological tissue, were manually excluded.

Two oral and maxillofacial pathologists (D.G.R. and S.S.S.N.) performed the ROI annotations, which were subsequently reviewed by two pathologists (A.L.D.A. and P.A.V.) to ensure consistency and diagnostic accuracy. Annotations were performed using the Aperio ImageScope software (Leica Biosystems, Wetzlar, Germany) with a digital drawing tablet (Huion Inspiroy H1060P) under standardized workstation conditions.

### Patch Extraction and Dataset Partitioning

Following ROI annotation, the selected regions were segmented and fragmented into fixed-size image patches of 224 × 224 pixels corresponding to the input dimensions of the EfficientNetV2-B0 [19], an architecture chosen based on the results obtained in the previous investigation conducted by our group [9], ensuring compatibility with the pre-trained architecture while preserving sufficient histological contexts. Each extracted patch inherited the class label of the annotated ROI in the corresponding WSI: class 0 (neurofibroma), class 1 (perineurioma), or class 2 (schwannoma).

To prevent data leakage and ensure independence between subsets, dataset partitioning was performed using a strict patient-wise split, ensuring that patches originating from the same patient were never distributed across different subsets. Although an approximate 80/10/10 split was initially targeted, this distribution could not be strictly achieved due to the patient-wise partitioning strategy described above. As a result, the final patch-level proportions deviate from the intended split, particularly for the perineurioma class. The final distribution of patches across training, validation, and test sets is summarized in Table 1. Validation and test sets consisted exclusively of original, non-synthetic patches.

**Table 1:** Classes in the patch division *per* subsets

	Total	Training	Validation	Test
Neurofibroma	42,590 (*n* = 9)	34,386 (81%) (*n* = 4)	4,175 (10%) (*n* = 3)	4,029 (9%) (*n* = 2)
Perineurioma	40,128 (*n* = 7)	25,690 (64%) (*n* = 5)	11,617 (29%) (*n* = 1)	2,821 (7%) (*n* = 1)
Schwannoma	103,563 (*n* = 14)	84,435 (82%) (*n* = 5)	9,258 (9%) (*n* = 5)	9,870 (10%) (*n* = 4)

### Generative Adversarial Network for Synthetic Image Generation

To address class imbalance and morphological heterogeneity within the perineurioma class, synthetic patches were generated using a Pix2Pix-inspired conditional Generative Adversarial Network (cGAN) with non-deterministic cross-patient pairing [20].

*Generator Architecture*: The generator followed an encoder–decoder structure without skip connections, enforcing compression of spatial information into a latent bottleneck representation within the encoder, capable of capturing global morphological patterns. Self-attention modules were incorporated to model long-range spatial dependencies within histological patches [21]. The network was trained using a combination of adversarial loss and L1 regularization term. Given that input–target pairs were constructed from patches derived from different patients, the L1 term does not represent a deterministic pixel-wise reconstruction objective, as in classical Pix2Pix frameworks. Instead, it acts as a structural regularizer that constrains the generator to preserve plausible histomorphological patterns while allowing variability between paired samples. Accordingly, the model should be interpreted as an adapted conditional GAN rather than a strict image-to-image translation framework, as the mapping between input and target images is not deterministic.

The GAN was trained using the Adam optimizer (learning rate = 2 × 10⁻⁴, β₁ = 0.5) for both generator and discriminator, with a batch size of 1. The total generator loss was defined as L_total = L_GAN + λ·L1, with λ = 100. Training was conducted for approximately 350,000 iterations, with model checkpoints saved every 5000 iterations. Input images were resized to 286 × 286 and randomly cropped to 256 × 256 (jittering augmentation), followed by normalization to the range [−1, 1]. Patches with excessive background or non-informative regions were excluded during preprocessing to ensure morphological relevance of training samples.

Synthetic image quality was assessed using the Structural Similarity Index (SSIM), which yielded a mean value of 0.63. This value indicates moderate structural similarity while preserving morphological variability, suggesting that synthetic images preserve relevant histomorphological patterns while maintaining variability; hence, without excessive similarity to original samples, thereby avoiding redundancy and supporting effective data augmentation. Importantly, values in this range are consistent with generative scenarios where exact pixel-wise correspondence is not expected, particularly in cross-patient pairing settings. Thus, SSIM in this context should be interpreted as an indicator of structural plausibility rather than reconstruction fidelity.

### Clinically-oriented Synthetic Image Generation Strategies

**Fig. 1:**
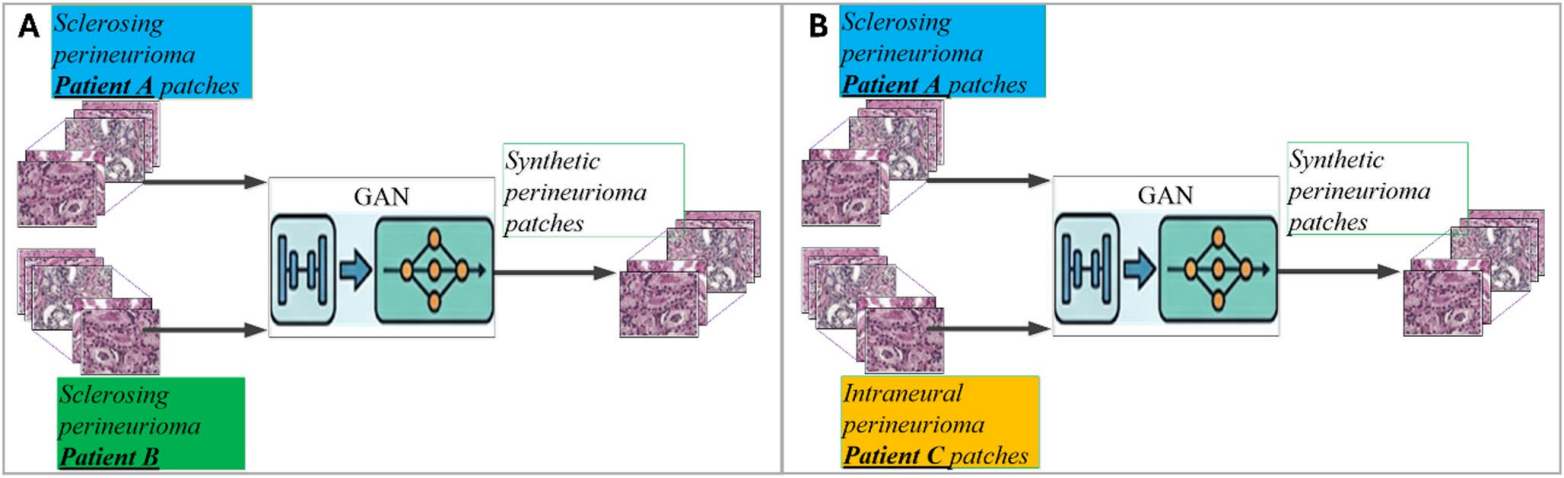
Morphology-driven synthetic generation strategies for perineurioma patches. Representative morphological variants (sclerosing and intraneural) serve as input to these strategies. **A**: Intra-phenotypic expansion: Patch pairing from two independent cases of sclerosing perineurioma to increase intra-class variability. **B.** Inter-phenotypic interpolation: Patch pairing between sclerosing and intraneural variants to model transitional morphological patterns. Synthetic images were used only in the training set; validation and testing were performed on original (non-synthetic) images

Two augmentation strategies were implemented (Fig. 1) to enrich the morphological variability of the perineurioma class, which represented the most heterogeneous and underrepresented category in our dataset. In both experiments, synthetic generation was restricted to patches derived from two training-set patients with perineurioma, allowing a controlled proof-of-concept evaluation of morphology-driven variability enrichment. In Experiment A, both patients corresponded to the sclerosing subtype, whereas in Experiment B the paired patients represented the sclerosing and intraneural variants, respectively.

#### Experiment A–Intra-phenotypic expansion (sclerosing × sclerosing)

In Experiment A, synthetic patches were generated by pairing patches from two independent training-set cases of sclerosing perineurioma included in the training subset. Let {*pA1*,* pA2*,*…*,* pAn*} denote the set of patches extracted from Patient A and {*pB1*,* pB2*,* …*,* pBm*} the set of patches from Patient B. Synthetic generation was performed by systematically sampling cross-patient patch pairs (e.g., *pA1×pB1*, *pA1×pB2*, …, *pAn×pBm*) as input conditioning instances for the Pix2Pix-based model. This design aimed to increase within-phenotype variability while remaining constrained to a single histological subtype, thereby encouraging the generator to recombine stromal density, collagenous backgrounds, and spindle-cell organization characteristic of sclerosing perineurioma (Fig. 1). The final output of this procedure yielded 11,542 synthetic perineurioma patches used exclusively in the training subset.

#### Experiment B–Inter-phenotypic interpolation (sclerosing × intraneural)

In Experiment B, synthetic patches were generated by pairing patches from two independent training-set cases representing distinct perineurioma variants with distinct architectural patterns (sclerosing and intraneural) to model transitional morphology. Using the same cross-patient pairing principle described above, patch pairs were sampled across the two phenotypic domains to drive cross-domain translation and generate intermediate representations. This strategy was designed to expose the classifier to a broader morphological spectrum, including hybrid patterns that may occur at the boundary between variants (Fig. 1). This procedure also yielded 11,542 synthetic perineurioma patches incorporated only into the training subset.

#### Rationale for synthetic volume and class targeting

The number of synthetic patches (11542 per strategy) was intentionally constrained by the practical opportunity to leverage available training cases in a controlled proof-of-concept setting, rather than aiming to fully equalize all classes to the largest category. The central objective of this study was to test whether biologically motivated morphological enrichment of the most challenging class (perineurioma) would improve downstream classification, given prior evidence of limited sensitivity for this entity when trained on original images alone. Although the neurofibroma class presented a comparable sample size, its classification performance was consistently high across all evaluated metrics. From a modeling perspective, this suggests that the class already exhibits sufficient morphological variability to support stable learning of decision boundaries. Therefore, it was not prioritized for synthetic augmentation in this study, which focused on addressing the performance gap observed in the perineurioma class. Generation of larger synthetic volumes (e.g., matching the largest class) and/or balanced multi-class synthetic augmentation across all three entities represents an important future direction, but was outside the scope of the present study, which focused on isolating the effect of perineurioma-specific variability enrichment under a fixed validation/testing protocol.

### Synthetic Patch Generation

**Fig. 2:**
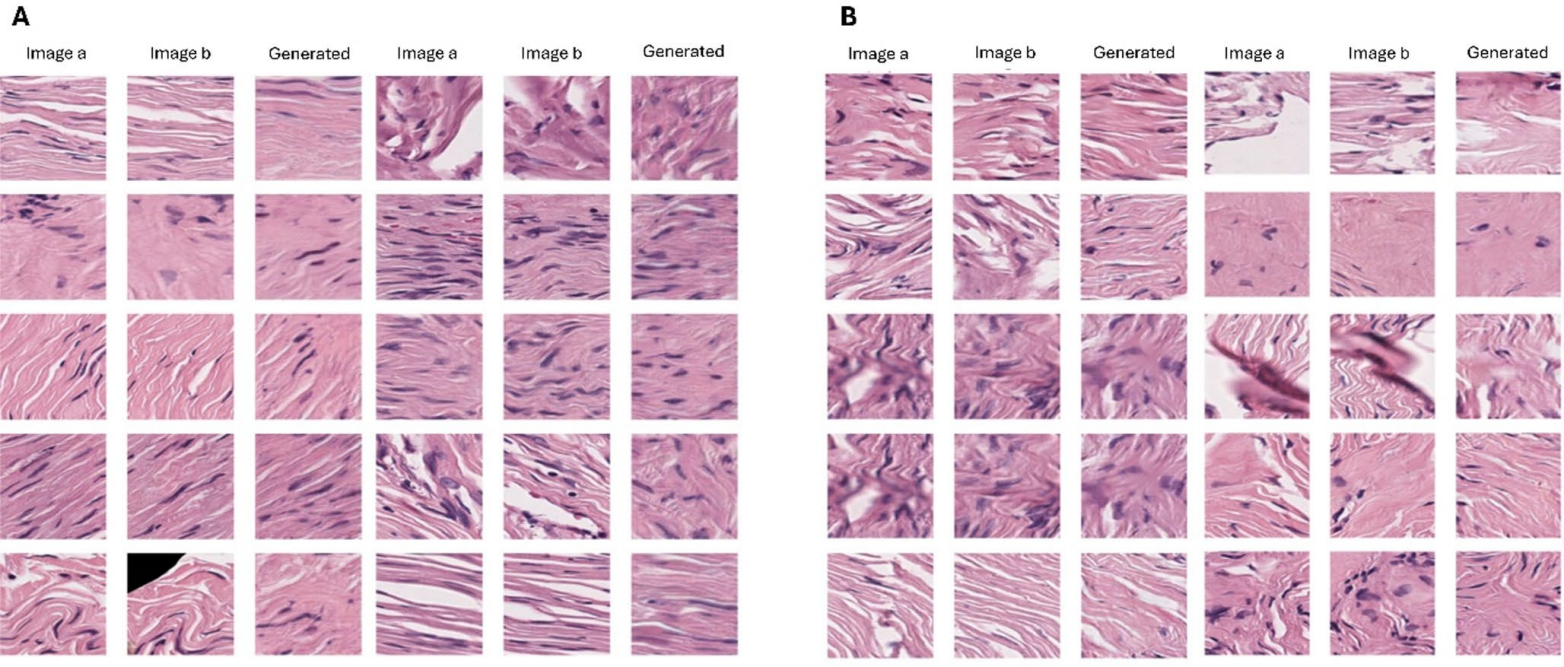
Examples of synthetic patch generation in experiments **A** and **B**. Pairs of real input patches (Image a and Image b) are used to generate a new synthetic sample (Generated). The resulting patches exhibit intermediate morphological characteristics while preserving overall histological coherence, illustrating the model’s ability to recombine structural patterns from the input data

To qualitatively illustrate the generative process, examples of synthetic patch creation are presented in Fig. 2. In this framework, pairs of real input patches (Image a and Image b) are used to guide the generation of a new synthetic patch (Generated), enabling the model to learn and combine morphological patterns observed in the input data. The generated patches exhibit intermediate structural characteristics between the input samples while preserving histomorphological coherence. This behavior suggests that the generative model is capable of capturing and recombining relevant tissue patterns, producing plausible variations that extend beyond simple replication of the original data. These synthetic samples were subsequently incorporated into the training set to evaluate their impact on feature space representation and classification performance.

### Feature Space Analysis

**Fig. 3:**
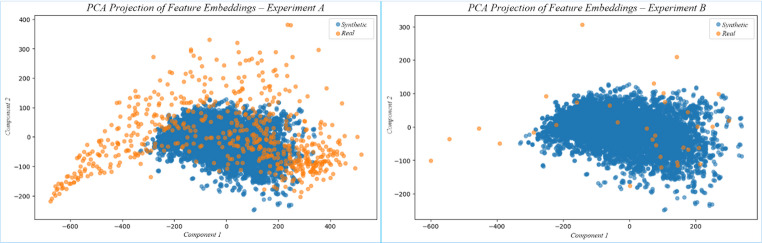
Principal Component Analysis (PCA) projection (first two components) of deep feature embeddings for real and synthetic patches. Synthetic samples partially overlap with real data while extending the feature space, suggesting preservation of core morphological characteristics with increased variability

To assess the distribution of synthetic and original patches, dimensionality reduction techniques were applied. Principal Component Analysis (PCA) was used to visualize feature distribution within a reduced-dimensional feature embedding space derived from deep representations [22] (Fig. 3). Synthetic samples demonstrated partial overlap with original patches while also expanding the feature space, suggesting that the generative process preserved core morphological characteristics while increasing variability. Notably, synthetic patches appeared to occupy regions of the feature space that were sparsely represented by real data, suggesting a potential interpolation-like effect that enhances coverage of the underlying morphological manifold. The PCA visualization suggests that synthetic samples expanded the feature space around underrepresented perineurioma patterns, potentially reducing local sparsity and contributing to the observed improvement in recall for this class. From a learning perspective, this effect may also mitigate class imbalance by reducing feature sparsity and promoting smoother decision boundaries for underrepresented classes, which is consistent with the observed improvement in perineurioma recall. This behavior is consistent with the notion that generative augmentation may contribute to a more continuous and densely sampled representation of histomorphological patterns.

### Classification Model and Training Protocol

*Classification model*: The classification task was performed using an EfficientNetV2-B0 [19] architecture initialized with ImageNet-pretrained weights [23]. The final classification layer was adapted to a three-class output (neurofibroma, schwannoma, and perineurioma). Input patches of 224 × 224 pixels were used, following standard preprocessing for EfficientNet models, including normalization to match ImageNet statistics.

Three training configurations were evaluated: (1) Original dataset only; (2) Original dataset + Experiment A synthetic patches; (3) Original dataset + Experiment B synthetic patches. All performance metrics were computed exclusively on the independent test set composed of original patches.

*Training protocol*: The model was trained using the Adam optimizer with an initial learning rate of 1 × 10⁻³. Training was conducted for a fixed number of epochs, with validation-based monitoring to ensure stable convergence. Data augmentation included random rotations, horizontal and vertical flips, and slight variations in brightness and contrast to improve robustness to staining variability.

*Class imbalance handling*: Given the inherent class imbalance, particularly for perineurioma, strategies were adopted to mitigate biased learning. The training process incorporated class balancing through controlled sampling of patches, ensuring adequate representation of minority classes during training. This approach was critical to avoid dominance of majority classes and to enable the model to learn discriminative features across all tumor types.

*Bias control and data leakage prevention*: To ensure robust evaluation, data splitting was performed at the patient level, preventing patches from the same patient from appearing in both training and testing sets. This strategy minimizes information leakage and provides a more realistic estimate of generalization performance.

*Evaluation metrics*: Model performance was evaluated using accuracy, balanced accuracy, precision, recall, specificity, F1-score, Cohen’s kappa, and Matthews correlation coefficient (MCC) [24–26]. Given the class imbalance, balanced accuracy and MCC were considered particularly relevant for assessing classification performance across all classes.

All experiments were implemented in Python 3.10 using PyTorch and trained on an NVIDIA GeForce RTX 4090 GPU.

## Results

### Global Performance

The primary objective of the experiments was to evaluate whether morphology-driven synthetic enrichment of the perineurioma class could improve CNN classification performance. Global classification metrics are presented in Table 2. Both GAN-based augmentation strategies improved overall accuracy, compared to the baseline model trained exclusively on original images. Overall, both synthetic augmentation strategies improved global classification performance while maintaining high specificity for the perineurioma class and stable specificity across other tumor categories.

The baseline model trained exclusively on original images achieved an accuracy of 0.733. Incorporation of synthetic patches improved global performance under both augmentation strategies. Intra-phenotypic expansion (Experiment A) increased accuracy to 0.768 (Δ = +3.5% points), while inter-phenotypic interpolation (Experiment B) achieved the highest overall accuracy (0.776, Δ = +4.3% points), indicating improved global classification performance with the inter-phenotypic augmentation strategy. Balanced accuracy improved from 0.687 in the baseline model to 0.750 in Experiment A and 0.743 in Experiment B, reflecting improved class-balanced performance after synthetic enrichment. Macro-F1 increased from 0.663 to 0.733 (Experiment A) and 0.735 (Experiment B). Weighted F1 followed a similar trend, reaching 0.775 in Experiment B. Multiclass agreement metrics also improved. Matthews correlation coefficient (MCC) increased from 0.585 in the baseline configuration to 0.647 in Experiment A, and 0.637 in Experiment B. Cohen’s kappa rose from 0.567 to 0.628 and 0.616, respectively, indicating enhanced multiclass discriminative correlation after morphology-driven synthetic augmentation.

**Table 2:** Comparative evaluation of global classification metrics for EfficientNetV2-B0 trained with original and morphologically enriched synthetic datasets

Metric	Original Images	^a^Experiment A	^b^Experiment B
Accuracy	0.733	0.768	0.776
Balanced Accuracy	0.687	0.750	0.743
Macro Precision	0.794	0.826	0.825
Macro Recall	0.687	0.750	0.743
Macro F1	0.663	0.733	0.735
Weighted F1	0.731	0.771	0.775
Mean Specificity	0.866	0.881	0.880
Cohen’s Kappa	0.567	0.628	0.616
MCC (Multiclass)	0.585	0.647	0.637

^a^Intra-phenotypic expansion (sclerosing × sclerosing)

^b^Inter-phenotypic interpolation (sclerosing × intraneural)

### Per-Class Performance and Rare Class Enhancement

The perineurioma class (Class 1), which represented the most morphologically heterogeneous and diagnostically challenging category in the dataset, demonstrated the most clinically relevant improvement. Baseline recall was 0.34, reflecting limited sensitivity for this rare entity. Experiment A increased recall to 0.51 (Δ = +17% points), while Experiment B reached 0.47. Importantly, specificity for the perineurioma class remained extremely high (≥ 0.997), indicating that the increase in sensitivity did not introduce additional false positives. The F1-score for Class 1 improved from 0.50 in the baseline model to 0.67 in Experiment A and 0.64 in Experiment B. These findings indicate that morphology-driven synthetic augmentation can enhance sensitivity for rare tumor entities while preserving high diagnostic specificity (Table 3).

**Table 3:** Class-level diagnostic performance of EfficientNetV2-B0 using original training data and GAN-based morphological enrichment strategies

Model	Class	Precision	Recall (Sensitivity)	Specificity	F1
Original	0	0.515	0.970	0.711	0.67
	1	0.960	0.340	0.997	0.50
	2	0.907	0.750	0.890	0.82
^a^Experiment A	0	0.590	0.990	0.781	0.73
	1	1.000	0.510	1.000	0.67
	2	0.865	0.750	0.840	0.80
^b^Experiment B	0	0.590	0.980	0.782	0.73
	1	1.000	0.470	1.000	0.64
	2	0.895	0.780	0.857	0.83

^a^Intra-phenotypic expansion (sclerosing × sclerosing)

^b^Inter-phenotypic interpolation (sclerosing × intraneural)

### Strategy Comparison

Although augmentation was applied exclusively to the perineurioma class, the two strategies impacted the model differently. Intra-phenotypic augmentation (Experiment A) yielded the highest balanced accuracy and the greatest improvement in rare-class sensitivity, primarily enhancing within-class representation. On the other hand, Inter-phenotypic interpolation (Experiment B) achieved the highest overall accuracy and competitive multiclass agreement metrics, influencing the structure of decision boundaries between classes, and resulting in more globally consistent predictions, although the highest multiclass correlation metrics (MCC and Cohen’s Kappa) were observed in Experiment A.

## Discussion

PNSTs of the H&N frequently present overlapping histopathological features, particularly between neurofibroma and schwannoma [1–4]. Among PNSTs, perineurioma remains diagnostically challenging due to its morphological heterogeneity and relative rarity in routine diagnostic series [2, 4]. This complexity is reflected in the baseline performance of the classifier trained exclusively on original images, which demonstrated reduced sensitivity for the perineurioma class, consistent with prior observations in neural tumor classification tasks [9]. Notably, even in our previous experiments with high-capacity architectures such as EfficientNetV2-B0, the maximum sensitivity achieved for perineurioma remained limited (≈ 34%), further supporting that the main bottleneck lies in insufficient morphological representation rather than model capacity.

The present study investigated whether GAN-based synthetic augmentation could improve classification performance in this setting. GANs have increasingly been applied in digital pathology to address data scarcity, stain variability, and class imbalance [12–17]. However, most prior investigations have focused on common tumor types or binary classification problems. The application of structured generative strategies to morphologically heterogeneous PNSTs has remained limited.

Both augmentation strategies evaluated in this study were associated with improved overall model performance compared to the baseline configuration. Importantly, improvements were observed while maintaining validation and test sets composed exclusively of original images and generated exclusively from patients not used for synthetic generation, reducing the risk of artificial inflation of performance metrics.

Intra-phenotypic expansion (Experiment A) produced the largest improvement in balanced accuracy and rare-class sensitivity. By increasing variability within the sclerosing perineurioma subtype through cross-patient recombination of histological patches, the model was exposed to a broader representation of stromal architecture and spindle-cell arrangements. Prior studies have demonstrated that synthetic augmentation can enhance classifier robustness when variability within underrepresented classes is enriched [14, 15]. Our findings support the notion that structured intra-class expansion may improve decision boundary stability in morphologically subtle entities.

Inter-phenotypic interpolation (Experiment B) yielded the highest overall accuracy and multiclass correlation metrics. Feature space interpolation strategies have been proposed as a means of modeling continuous morphological transitions between phenotypic poles [18]. By generating transitional representations between sclerosing and intraneural variants through cross-phenotype patch pairing, this strategy may have facilitated generalization across the morphological spectrum of perineurioma.

These findings suggest that synthetic data generation may function not merely as a balancing mechanism but as a structural modeling tool capable of reshaping feature-space representations. Rather than simply increasing dataset size, augmentation strategies may alter the geometry of learned representations, enabling improved discrimination within biologically heterogeneous tumors.

From a diagnostic perspective, the improvement in perineurioma sensitivity without reduction in specificity is particularly relevant. False-positive rates remained consistently low across experiments, indicating that augmentation did not induce class boundary instability. Given that perineurioma frequently requires immunohistochemical confirmation in equivocal cases [5], improved computational sensitivity may support diagnostic triage or second‑opinion workflows in digital pathology. We acknowledge that GLUT‑1 and other IHC markers are effective for confirming perineurioma; however, IHC panels are time‑consuming and add cost. A computational model that achieves high specificity (≥ 0.997 for perineurioma) could serve as a pre‑screening tool to prioritize which cases require confirmatory IHC, thereby reducing unnecessary staining in morphologically straightforward cases. Furthermore, the primary contribution of this study is methodological: demonstrating that morphology‑driven GAN augmentation can improve classification of a heterogeneous, underrepresented entity, an approach that may extend to other rare tumors where IHC panels are limited or non‑specific.

Although GAN-based augmentation has shown promise in histopathology datasets [12–16], few studies have specifically addressed soft tissue tumors of the H&N with subtle architectural overlap. Our results extend the application of generative modeling to this diagnostically nuanced tumor group and demonstrate measurable improvement under a strictly controlled evaluation framework.

Limitations warrant consideration. First, the dataset size remains moderate, reflecting the rarity of perineurioma. In addition, synthetic generation was intentionally restricted to the perineurioma class to evaluate the impact of targeted morphological enrichment rather than full class balancing. Second, synthetic image quality assessment was limited to SSIM and feature-space visualization, without formal expert-based validation of morphological realism. Third, performance metrics were computed at the patch level. Although a strict patient-wise split was enforced to prevent data leakage, multiple patches derived from the same biological sample exhibit high structural and intra-case correlation and are not statistically independent observations. Estimating confidence intervals directly at the patch level would incur a pseudo replication bias, artificially inflating the degrees of freedom, underestimating uncertainty, and overstating statistical robustness. Given the pilot nature of this study and the restricted patient-level sample size (*n* = 30, reason for the proposed approach), robust patient-level resampling (e.g., patient-centric bootstrapping or repeated K-fold cross-validation) was computationally unstable and mathematically inappropriate. Future studies involving larger multicenter cohorts should incorporate patient-level aggregation and statistical uncertainty estimation. Finally, external validation using independent institutional cohorts was not performed, since the primary objective of this study was to evaluate the methodological impact of morphology-driven synthetic augmentation under controlled experimental conditions. Therefore, while our findings suggest only a modest overall enhancement in model performance through GAN‑based augmentation, the clinical significance of this observation must be viewed with caution. This study should be considered a proof‑of‑concept pilot investigation. Nevertheless, from a clinical screening perspective, the improvement in perineurioma recall (from 0.34 to 0.51) is noteworthy: even a modest absolute gain can meaningfully reduce false negatives for rare diagnostic categories, thereby supporting more robust and sensitive models in daily pathology workflows. Future studies should systematically evaluate the effect of varying synthetic-to-real patch ratios (e.g., 1:1, 2:1, 5:1, 10:1) on classification performance, as the optimal balance between real and GAN-generated data remains unknown for rare, morphologically heterogeneous tumors. Beyond ratio optimization, future investigations should also incorporate patient-level aggregation and multicenter datasets to further evaluate clinical applicability, as well as interpretability analyses, such as saliency mapping or region-level attribution, to better characterize the morphological features driving classification decisions [6, 7].

## Conclusion

GAN-based synthetic augmentation improved the classification performance of EfficientNetV2-B0 in distinguishing PNSTs. Both intra-phenotypic expansion and inter-phenotypic interpolation enhanced diagnostic metrics compared to training on original images alone, consistent with prior reports demonstrating the utility of generative models in histopathological data augmentation.

Intra-phenotypic augmentation yielded the strongest improvement in balanced accuracy and sensitivity for perineurioma, a morphologically heterogeneous and relatively underrepresented entity. In contrast, inter-phenotypic interpolation achieved the highest overall multiclass agreement, supporting the hypothesis that modeling transitional morphological patterns may enhance feature-space generalization.

These findings reinforce the potential role of structured and biologically informed generative augmentation strategies as complementary tools in digital pathology pipelines, particularly for rare or architecturally heterogeneous tumor entities where conventional datasets may be insufficient to support robust deep learning models.

## Data Availability

The datasets used and/or analyzed during the current study are available from the corresponding author on reasonable request. All authors agree to be accountable for any aspects of the work and we ensure that questions related to the accuracy or integrity of any part of the work are appropriately investigated and resolved.

## Code Availability

The code used for data preprocessing, model training, evaluation, and visualization in this study are available from the corresponding author on reasonable request.
